# Mechanical properties of silk of the Australian golden orb weavers *Nephila pilipes* and *Nephila* *plumipes*

**DOI:** 10.1242/bio.029249

**Published:** 2018-02-01

**Authors:** Genevieve G. Kerr, Helen F. Nahrung, Aaron Wiegand, Joanna Kristoffersen, Peter Killen, Cameron Brown, Joanne Macdonald

**Affiliations:** 1 Genecology Research Centre and School of Science, Engineering and Education, University of the Sunshine Coast, Sippy Downs, Queensland 4556, Australia; 2 Botnar Research Centre, Nuffield Department of Orthopaedics, Rheumatology and Musculoskeletal Sciences, University of Oxford, Oxford, OX3 7LD, UK; 3 Institute of Health and Biomedical Innovation and School of Chemistry, Physics and Mechanical Engineering, Queensland University of Technology, Brisbane, Queensland 4000, Australia; 4 Division of Experimental Therapeutics, Columbia University, New York, NY 10032, USA

**Keywords:** *Nephila* sp, Spider silk, Toughness, Stress-strain

## Abstract

Silks from orb-weaving spiders are exceptionally tough, producing a model polymer for biomimetic fibre development. The mechanical properties of naturally spun silk threads from two species of Australian orb-weavers, *Nephila pilipes* and *Nephila* *plumipes*, were examined here in relation to overall thread diameter, the size and number of fibres within threads, and spider size. *N. pilipes*, the larger of the two species, had significantly tougher silk with higher strain capacity than its smaller congener, producing threads with average toughness of 150 MJ m^−3^, despite thread diameter, mean fibre diameter and number of fibres per thread not differing significantly between the two species. Within *N. pilipes*, smaller silk fibres were produced by larger spiders, yielding tougher threads. In contrast, while spider size was correlated with thread diameter in *N. plumipes*, there were no clear patterns relating to silk toughness, which suggests that the differences in properties between the silk of the two species arise through differing molecular structure. Our results support previous studies that found that the mechanical properties of silk differ between distantly related spider species, and extends on that work to show that the mechanical and physical properties of silk from more closely related species can also differ remarkably.

## Abstract (precis)

**Summary:** Spider silk is a remarkably tough and extensible biomaterial. We identified macrostuctural and mechanical differences in outer web frame silk of two Australian orb-weaving spiders.

## INTRODUCTION

Spider silk is mechanically outstanding: its toughness (amount of energy per unit volume absorbed before rupture) exceeds that of the best synthetic high-performance fibres, including steel and Kevlar (Agnarsson et al., 2010; Guthold et al., 2007; Omenetto and Kaplan, 2010), due to its combination of strength and extensibility (Heim et al., 2009; Rising et al., 2005; Vendrely and Scheibel, 2007; Vollrath and Porter, 2006). While there are many types of silk, the Major Ampullate (MA) silk produced by orb-weaving spiders is exceptionally strong, extensible and tough, producing silk as tough as 111 MJ m^−3^ [*Nephila clavipes* (Linnaeus) *–* Nephilidae] and 354 MJ m^−3^ (*Caerostris darwini* Kuntner and Agnarsson *–* Araneidae) (Agnarsson et al., 2010).

Fewer than 50 spider species (of ∼40,000) have had their silk macrostructure and mechanical characteristics analysed (Agnarsson et al., 2010). MA silk from the golden orb weaver *N. clavipes* is the most extensively characterised, and has helped unveil the molecular architecture of spider silks. Recent reports comparing silk stress-strain properties for different spider species (Agnarsson et al., 2010) suggest that further studies should be modelled from tougher silks (Jastrzebska et al., 2014; Vollrath, 2000), because characterisation of a greater variety of threads should improve our molecular understanding of their mechanical properties. Here, we characterised the outer web frame, comprising bundles of MA silk fibres, from two species of Australian golden orb weavers, *Nephila pilipes* (Fabricius) and *Nephila* *plumipes* (Latreille). *N. pilipes* is one of the largest orb-weaving spiders (Su et al., 2007), altering its dragline silk protein in response to variation in prey (Tso et al., 2005), while *N. plumipes* is a smaller Australian species. Both species are diurnal, constructing large, asymmetric orb-webs which they occupy permanently, and sometimes capture prey up to several times larger and heavier than themselves (Harvey et al., 2007; Nyffeler and Knörnschild, 2013). Except when gravid, resident females repair webs within 10–60 min of damage, but will consume and rebuild, or relocate if damage is severe, repeated, or prey capture scarce (Harvey et al., 2007). Both species are widespread in north-eastern and northern coastal Australia, but *N. pilipes* is genetically divergent from its congeners within Australia (Harvey et al., 2007).

We hypothesised that *N. pilipes* would display greater mechanical capabilities than *N. plumipes* because of its larger size. We reasoned that, evolutionarily, this spider may require stronger silk to support its weight – both in terms of its heaviness on the web, and a requirement to catch sufficient prey to nutritionally support its large size (Guthold et al., 2007; Tso et al., 2005). This complements studies by Sensenig et al. (2010), who examined relationships between different spider species, sizes, and web architecture and quality (Sensenig et al., 2010). We thus compared the tensile strength of *N. pilipes* and *N. plumipes* dragline silk. In addition, we studied the relationship between toughness and thread morphology by comparing macrostructure (total thread diameter, diameter and number of fibres) and mechanical (stress, strain, yield and toughness) properties of dragline silk of the two species.

## RESULTS

### Silk and spider characteristics

**Fig. 1.**
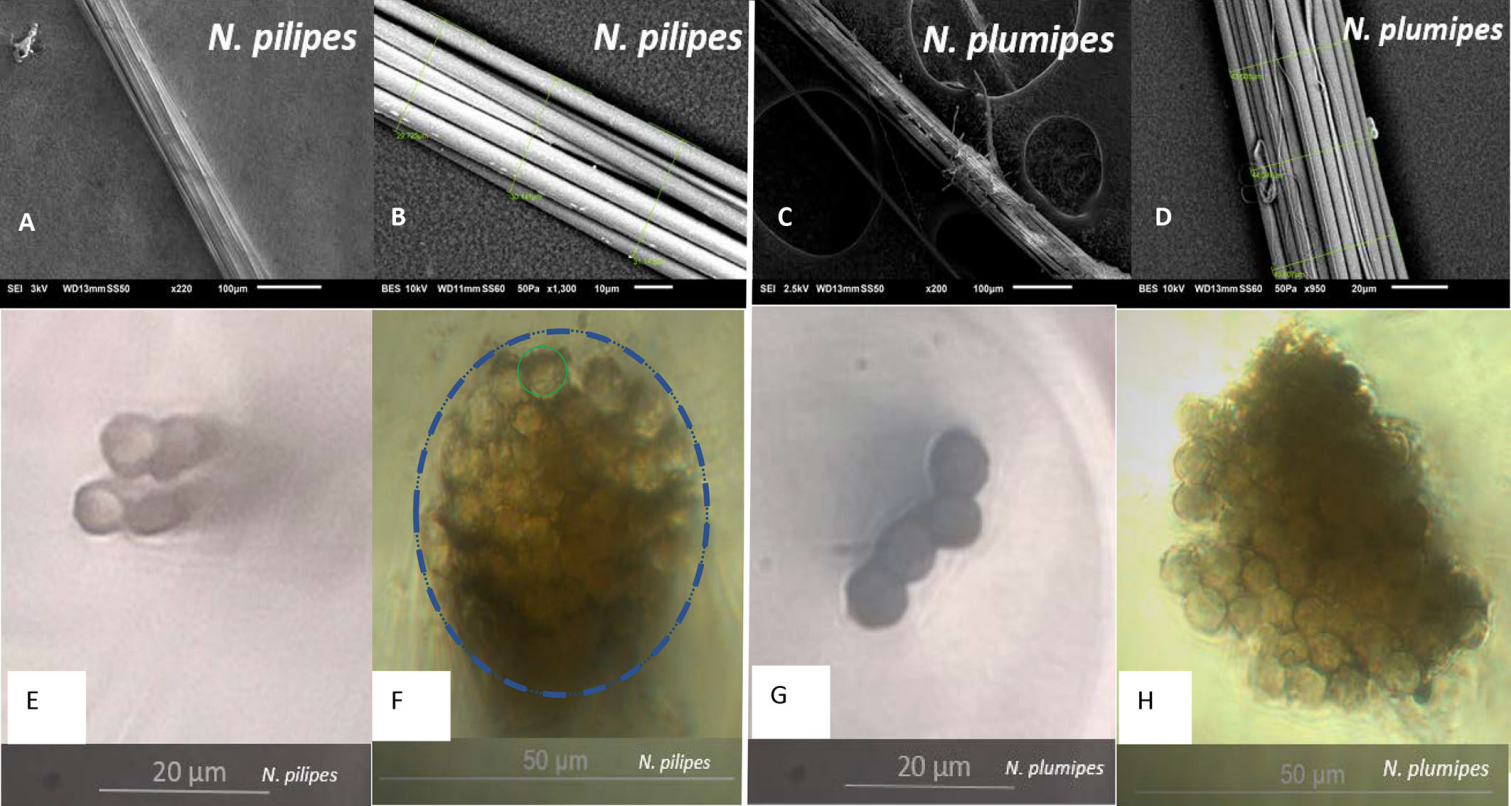
**Silk threads from *N. pilipes* (left) and *N. plumipes* (right) viewed under a confocal microscope (bottom) and by SEM (top).** (A,C) SEM image of threads at 200/220× magnification; (B,D) closer magnification displaying silk thread diameter measurements; (E-H) cross-sections of threads viewed under a confocal microscope showing the variation in fibre numbers per thread (fewer fibres, E,G; larger fibre numbers, F,H). The thread in F is designated with a blue dotted line, and the green solid line circle indicates a single fibre.

Female *N. pilipes* were significantly larger than *N. plumipes*, but their outer web frame threads did not differ in overall size, nor in individual fibre size or number (Table 1, Fig. 1).

**Table 1.**
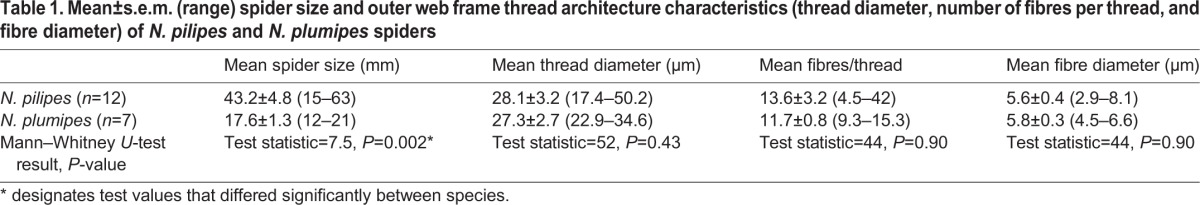
**Mean±s.e.m. (range) spider size and outer web frame thread architecture characteristics (thread diameter, number of fibres per thread, and fibre diameter) of *N. pilipes* and *N. plumipes* spiders**

### Silk mechanical properties

**Fig. 2.**
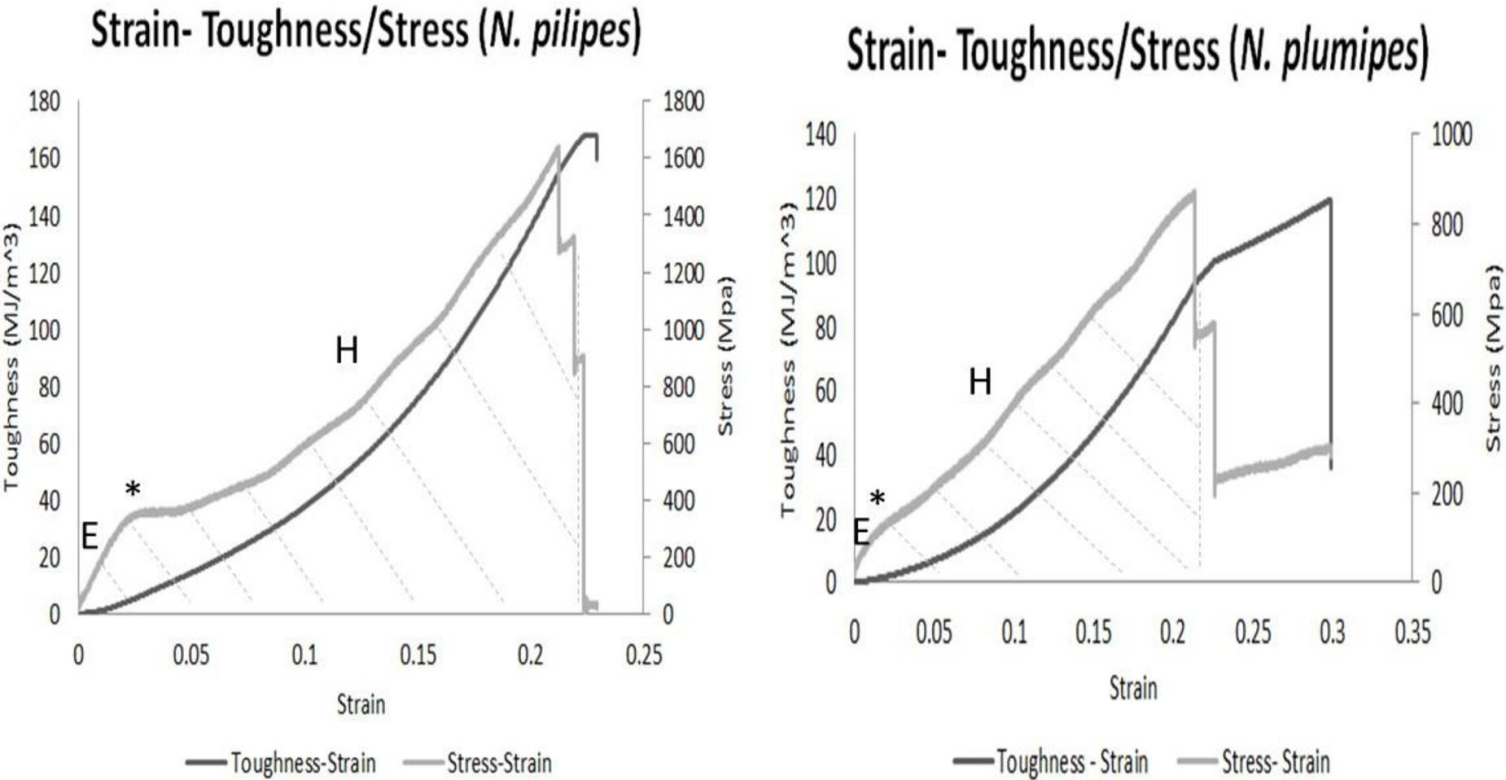
**Example stress-strain curves in relation to toughness for single threads of *N. pilipes* (left) and *N. plumipes* (right) thread.** Silk was pulled at 1 mm s^−1^. Stress-strain curves have distinct regions signifying behavioural and structural change: before the yield point (*) the response is elastic and the curve is straight. This first slope is the initial elastic modulus (E). The yield point marks the transition between an elastic and rubber-like response. It is assumed that the amorphous fraction converts from a glass state to a rubber state at this point (Gosline et al., 1999). The gradient of the stress-strain curve falls at the yield point altering E, followed by an increase in slope (and E) as the strain continues to increase, known as work hardening (H). Post-yield response is due to behaviour of action between the rubber states and the crystalline fractions of the silk. Immediately following yield, the stiffness is due to the rubber fraction, but as strain increases and the polymer chains are forced together by increasing tensile strain, the rubber states convert to either glass or crystal, giving a stiffer material that ultimately breaks with a brittle response (Gosline et al., 1999). Points of decreased stress indicate fibre breakage with individual fibres fracturing at different points in time. Stress, strain and toughness were measured from the first fracture, indicated by the hatched area.

Of the individual threads tested for *N. pilipes* (*n*=34 from 12 spiders) and *N. plumipes* (*n*=27 from seven spiders), 28% gave invalid results from mechanical failure or fracturing at the attachment site instead of the thread's centre and were discarded from analyses. This resulted in final data calculated from 25 and 19 threads (Fig. 2), for which replicate threads were averaged for each spider.

Silk threads of *N. pilipes* were significantly tougher and withstood significantly greater strain until fracture than threads of *N. plumipes*; ultimate tensile strength (UTS) and yield strength, however, did not differ between the two species (Table 2).

**Table 2.**
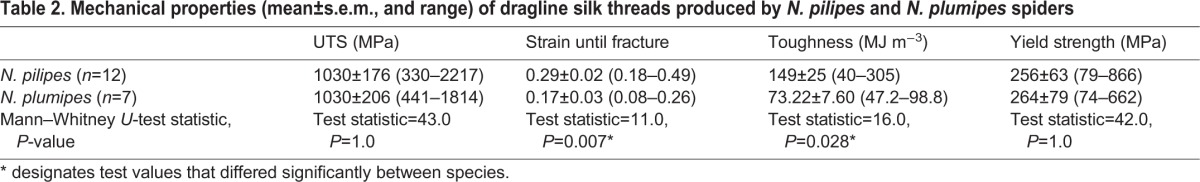
**Mechanical properties (mean±s.e.m., and range) of dragline silk threads produced by *N. pilipes* and *N. plumipes* spiders**

Spearman's rank correlation was used to identify possible correlations between data sets, with *P*<0.05, demonstrating rejection of the null hypothesis that the samples were unrelated, and Spearman's rho values (ρ), illustrating the strength and direction of the relationship (Tables 3 and 4). Thread diameter was positively correlated with the number of fibres for *N. pilipes* (ρ=0.80, *P*=0.002) but not for *N. plumipes* (ρ=0.36, *P*=0.43). Overall, larger *N. plumipes* females produced larger diameter threads (ρ=0.88, *P*=0.009), while larger *N. pilipes* females produced smaller diameter fibres (ρ=−0.73, *P*=0.007). Fibre diameter in *N. pilipes* was negatively correlated with thread toughness (ρ=−0.87, *P*<0.001), yield (ρ=−0.81, *P*=0.007) and UTS (ρ=−0.93, *P*<0.001). Within *N. pilipes*, spider size was positively correlated with silk toughness (ρ=0.76, *P*=0.004) and yield (ρ=0.61, *P*=0.037), producing a non-collinear relationship between size, toughness and fibre diameter. *N. plumipes*, however, showed no statistical relationships between thread toughness and any measured parameter.

**Table 3.**
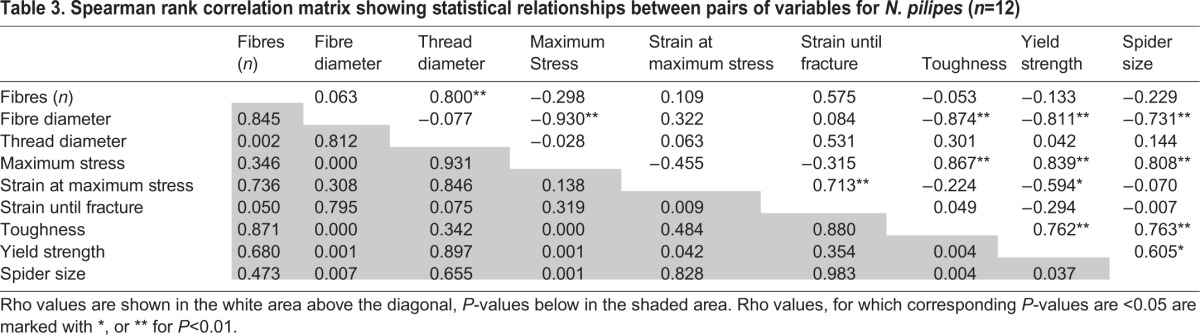
**Spearman rank correlation matrix showing statistical relationships between pairs of variables for *N. pilipes* (*n*=12)**

**Table 4.**
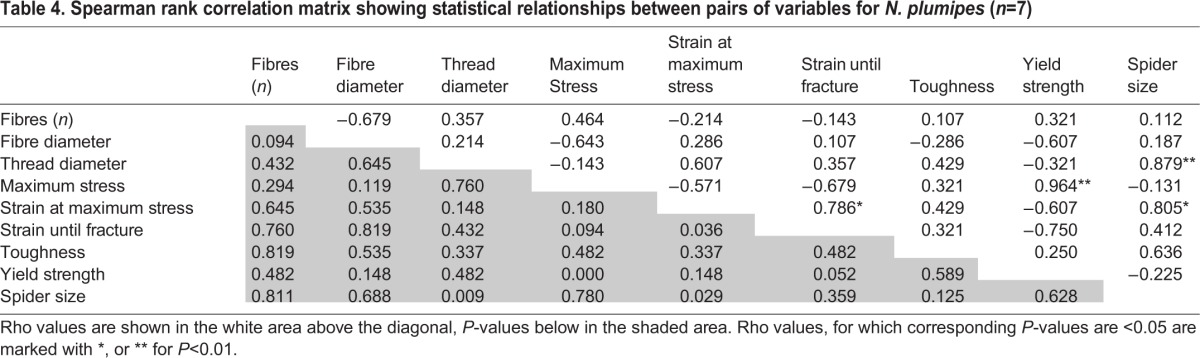
**Spearman rank correlation matrix showing statistical relationships between pairs of variables for *N. plumipes* (*n*=7)**

## DISCUSSION

The mechanical properties of spider silk are controlled by the mechanisms of energy storage and dissipation from the molecular to macroscopic level (Brown et al., 2012, 2011; Cranford et al., 2012; Dunaway et al., 1995; Nova et al., 2010). Here, we explored the effect of fibre level properties of silk for two Australian golden orb spiders, the large rainforest species, *N. pilipes*, and its smaller congener, *N. plumipes*. We hypothesized that the larger spider would produce a tougher silk, to support the additional weight of the spider on the web and to assist with catching sufficient prey to nutritionally support its large size (Guthold et al., 2007; Vollrath, 1999). We also chose to study outer web frame silk, which is reinforced by the spider and not commonly studied, to provide a unique view of silk when used naturally by the spider. Our results confirmed that the silk of *N. pilipes* was significantly tougher than that of *N. plumipes.* While not directly comparable to radial silk (which consists of two fibres), we observed that silk from *N. plumipes* produced unexceptional toughness properties, whereas the larger-bodied *N. pilipes* produced some fibres that were above average toughness for spiders, based on the previously reported average toughness of 107 MJ m^−3^ (Agnarsson et al., 2010).

Interestingly, by using outer web frame, we were able to observe a greater number of fibres present within a silk thread than is normally studied. We determined that *N. pilipes* had a smaller fibre diameter, and this fibre diameter inversely correlated to silk toughness and size of the spider. The observed relationship between spider size, fibre size and mechanical performance for *N. pilipes* corresponds with previous observations that an (artificial) increase in spider weight and size increased the silks' fibre diameter, thus decreasing silk toughness (Vollrath and Kohler, 1996). Similarly, another study demonstrated that larger species of spiders produce a higher quality material, improving web performance (Sensenig et al., 2010). However, *Caerostris darwini* averages only 20 mm in size and yet produces exceptionally tough silk (averaging 350 MJ m^−3^) (Agnarsson et al., 2010), presumably to withstand the weight of its large web (which includes anchor threads spanning up to 25 m in diameter) (Gregorič et al., 2011). In our own study, while *N. plumipes* showed a positive relationship between body size and fibre size, there was no corresponding relationship between fibre size and silk toughness. In contrast, *N. pilipes* could optimise fibre diameter (and toughness) in relation to its body size*.*

The *N. pilipes* correlation between decreasing silk fibre diameter and increasing toughness is supported by previous theoretical analysis, indicating that only silk threads with smaller diameters display exceptional resistance to failure and deformation (Giesa et al., 2011), due to a synergistic relationship between the silk fibrils and fibres (Fu and Lauke, 1996; Giesa et al., 2011; Jelinski, 1998). In addition, Swanson et al. (2007) conducted a comparative study of capture silk among several orb-weaving spider species, and found that those which spin small diameter fibres tend to have tougher silk, suggesting compensation to maintain total breaking energy of the thread. They also found a negative relationship between strength and extensibility across species, indicating a potential evolutionary trade-off. We found a similar correlation for dragline silk in *N. pilipes* and a trend towards this in *N. plumipes*: the balance between strength and extensibility endows enormous toughness (and a high level of internal molecular friction) to MA silk, the function of which is to support the web and its contents, and absorb the kinetic energy of impacting prey (Sensenig et al., 2012).

Interestingly, the reinforced nature of the outer web frame did not contribute to additional toughness, as larger number of fibres within a thread had no effect on silk toughness in either *N. pilipes* or *N. plumipes*, suggesting that fibres within an outer web frame thread act independently, or with only weak interaction. This is contrary to expectation in an otherwise highly optimised material, in which standard engineering approaches such as fibre twist ensure strong contact, variance of fibre strength (here, by diameter) and therefore suppression of critical fracture clusters, or the use of higher numbers of smaller fibres might be anticipated. The absence of these features in spider outer web frame thread mechanics are somewhat dispiriting for the engineer, as such commonly used toughening mechanisms appear to have been determined inefficient by the evolutionary process.

It appears, therefore, that the main mechanism underpinning the different mechanical properties of *N. plumipes* and *N. plumipes* is at the level of protein structure. This is supported by the lack of relationship found between fibre size and toughness for *N. plumipes.* Despite its inferior performance, *N. plumipes* had similar numbers of fibres per thread and similar fibre diameters. Key measurements of fibre structure, such as crystallinity, crystal size, and alignment, were not made in this study. We note that as the size of the β-sheet nano-crystallite structure reduces, toughness of the fibre has been shown to increase (Du et al., 2006; Termonia, 1994). However, β-sheet crystallite conformation can vary substantially in relation to the speed at which silk is drawn from the spider's spinneret (Holland et al., 2012; Vollrath et al., 2001), and silk forcibly extracted can be weaker than natural silk due to alteration in the density of β-sheet crystallites (Agnarsson et al., 2010; Madsen and Vollrath, 2000). In addition, varying diets and available prey can similarly alter silk toughness, affecting the relative quantity of two key proteins in MA silk (Spidroin 1 (*MaSp1*) and Spidroin 2 (*MaSp2*) (Blamires et al., 2010; Blamires et al., 2016; Tso et al., 2005). To obtain ecologically relevant measurements for *N. pilipes* and *N. plumipes* outer web frame toughness, we collected freshly spun threads from spiders in their natural habitat. Our results, however, do not preclude that other ecological factors that could affect silk structure and toughness, such as spider age, body condition, ontogenetic stage, climate (including humidity), and prey; these could be interesting to investigate further.

In summary, our results indicated that the silk of *N. pilipes* was significantly tougher than that of *N. plumipes*, producing fibres that were above average toughness for spiders. We also determined that *N. pilipes* had a smaller fibre diameter, and this fibre diameter inversely correlated to silk toughness and size of the spider, but we did not identify a relationship between fibre size and silk toughness for *N. plumipes*. Closely related orb-weaving species vary substantially in the mechanical properties of their silk (Swanson et al., 2007), even in spiders from similar habitat. It is therefore not surprising that we found differences between two phylogenetically distant *Nephila* species. Overall, our results suggest that the differences observed between *N. plumipes* and *N. pilipes* silk are likely due to differences in the underlying molecular structures within their fibres.

## MATERIALS AND METHODS

### Spider silk collection

Spider silk was collected between June 2015 and January 2016, within a 10 km radius of Buderim, Queensland, Australia. *N. pilipes* webs were collected from native habitat, often from isolated areas within subtropical forests, no closer than 10 m from each other. *N. plumipes* webs were collected in disturbed areas, including backyards, planted forests and farmlands. *N. plumipes* were commonly found in groups, with inter-connected webs, with up to 15 spiders within a 10 m radius (G.G.K, personal observation).

Bundles of silk fibres (major ampullate) were taken from the frame of each web in natural habitat to obtain ecologically relevant measurements. While many studies are performed on radial web sections, which contain only two fibres (e.g. Agnarsson et al., 2010), we used frame silk because spiders often reinforce this web section with multiple threads. The higher numbers of fibres, and consequently higher forces, also reduces error from noise in force measurements. Twelve *N. pilipes* and seven *N. plumipes* webs were tested. Webs were disturbed at around 16:00 by removing outer frame threads to ensure the spider repaired this section of the web with fresh silk. At approximately 09:00 the following day, the fresh silk from this web region was fixed using micropore tape on to a 250×165 mm collection grid in 200 mm, 20 mm and 10 mm sections for tensile testing, scanning electron microscopy (SEM), and confocal microscopy, respectively. The silk was glued to the grid at its natural tension, cleaved from the web, and stored in a humid airtight container for up to 3 days prior to testing. The resident female spider's size (top of head to end of abdomen) was measured using a ruler (±1 mm). Only adult female spiders were used. Cross-sectional images of each thread were taken using a confocal microscope, and images and measurements were made using SEM.

### Silk and spider physical characteristics

The 10 mm collected silk sections were embedded into an epoxy resin and set for 48 h. Mounted samples were cut and viewed under a confocal microscope (Nikon Eclipse Ti-E, Nikon, Tokyo, Japan) at 400× magnification. The number of fibres within each thread was counted, and the radial diameter of every fibre was measured. This was used to determine cross-sectional area of sections from the outer web frame thread, from the sum of each individual fibre cross-sectional areas.

The 20 mm collected silk thread sections were mounted onto carbon stubs and immediately examined uncoated using a JSM-6610 SEM (JEOL Ltd., Tokyo, Japan) in low vacuum mode at 1-3 kV. The number of fibres within each thread was counted, and the diameter of each fibre was measured (±1 µm) at 200–1000× magnification. Thread diameter was similarly measured 3–6 times along the sample, averaged, and used to estimate silk volume for subsequent toughness calculations.

Two to four threads were measured for each spider. Spider size, thread diameter, and the number and diameter of individual fibres were compared between the two species using a Mann–Whitney *U*-test because data did not conform to assumptions for parametric testing.

### Silk mechanical properties

A low-speed tensile test was configured to establish stress-strain curves and calculate toughness for individual threads. Thread from the 200 mm collecting grid was fastened to a F329 1 N load cell (Novatech, St Leonards on Sea, UK), and thread length at zero tension was recorded. The silk was then extended at 1 mm/s using a Rotary Motion Sensor (PASCO, Roseville, CA, USA) until breakage. The angular position of the spindle, length of silk, and tension (measured by load cell) was recorded as a function of time using Data studio software (1.9.8.10, PASCO), which were then used to determine the stress and corresponding strain of the silk. Toughness is the area under the force x displacement curve divided by initial volume, where the area was only measured from the silk's first point of fracture (Fig. 2). Engineering stress was recorded. Toughness, strain until fracture, UTS and yield strength were compared between the two spider species using Mann–Whitney *U*-tests. Within each spider species, Spearman rank correlations were conducted to examine relationships between spider and silk macrostructure characteristics and mechanical properties. The statistics software used was IBM SPSS Statistics V22.
